# A Novel Non-Alcoholic Einkorn-Based Beverage Produced by Lactic Acid Fermentation: Microbiological, Chemical, and Sensory Assessment

**DOI:** 10.3390/foods13233923

**Published:** 2024-12-04

**Authors:** Antonietta Maoloni, Martina Cirlini, Lorenzo Del Vecchio, Raquel Torrijos, Eleonora Carini, Giorgia Rampanti, Federica Cardinali, Vesna Milanović, Cristiana Garofalo, Andrea Osimani, Lucia Aquilanti

**Affiliations:** 1 Dipartimento di Scienze Agrarie, Alimentari, e Ambientali, Università Politecnica delle Marche, via Brecce Bianche, 60131 Ancona, Italy; a.maoloni@univpm.it (A.M.); g.rampanti@univpm.it (G.R.); f.cardinali@univpm.it (F.C.); v.milanovic@univpm.it (V.M.); c.garofalo@univpm.it (C.G.); a.osimani@univpm.it (A.O.); 2 Food and Drug Department, University of Parma, Parco Area delle Scienze 27/A, 43124 Parma, Italy; martina.cirlini@unipr.it (M.C.); lorenzo.delvecchio@unipr.it (L.D.V.); raquel.torrijos@uv.es (R.T.); eleonora.carini@unipr.it (E.C.); 3 Laboratory of Food Chemistry and Toxicology, Faculty of Pharmacy, University of Valencia, Ave. Vicent Andrés Estellés s/n, 46100 Burjassot, Spain

**Keywords:** multiple-strain starter, lactic acid bacteria, lactic acid fermentation, cereal-based yogurt-like fermented beverage, chemical evolution

## Abstract

Einkorn (*Triticum monococcum* L. ssp. *monococcum*) is gaining renewed interest for its high nutritional value and digestibility. Lactic acid fermentation could enhance these properties by improving micronutrient bioavailability, sensory properties, and shelf life. This study aimed to develop a novel non-alcoholic einkorn-based beverage through lactic acid fermentation. A multiple-strain starter was selected based on acidifying properties and inoculated into an einkorn-based substrate to produce a yogurt-like beverage. Prototypes were evaluated through physico-chemical, chemical, and microbiological analyses and compared to uninoculated controls. A sensory analysis was also performed to assess flavor attributes before and after lactic acid fermentation. The inoculated starter culture reached a load of approximately 9 Log CFU g⁻¹ and remained viable throughout storage, leading to an increase in lactic acid concentration and high titratable acidity, corresponding to low pH values. Total polyphenol content increased during fermentation and remained stable during storage, whereas antioxidant activity did not show significant differences over time. An increase in monosaccharides, acids, and ketones was observed during fermentation and storage. The prototypes exhibited a distinctive proximate composition, along with yogurt and fruity aroma notes. These results suggest the feasibility of producing a safe and stable non-alcoholic einkorn-based fermented beverage with appealing sensory characteristics.

## 1. Introduction

For millennia, einkorn (*Triticum monococcum* L. ssp. *monococcum*) served as a staple food for early farmers. However, its production significantly decreased in the 1960s, and today its cultivation is primarily concentrated in specific regions of the Mediterranean basin and continental Europe. The renewed interest in this crop is largely due to its ability to thrive in low-input agricultural systems, its strong resistance to pests and diseases, and its superior nutritional quality compared to polyploid wheat varieties [1]. This last aspect is closely linked to the increasing consumer demand for functional foods and those with high nutritional value, making it a key factor in the development of innovative, consumer-attractive food products [2,3,4]. While einkorn wheat generally causes milder reactions compared to other *Triticum* species, it is important to note that it remains unsuitable for individuals with celiac disease [2,5].

Einkorn whole meal has a low dietary fiber content but is rich in protein, lipids (primarily unsaturated fatty acids), fructans, and essential trace elements such as zinc and iron [2]. In addition, einkorn is a cereal with very high biological value and digestibility. The former is primarily attributed to a high concentration of various antioxidant compounds, including carotenoids, tocols, conjugated polyphenols, alkylresorcinols, and phytosterols. Additionally, its low β-amylase and lipoxygenase activities are recognized for minimizing the degradation of antioxidants during processing [2,3,6,7,8]. The higher digestibility of einkorn compared to wheat and other cereals is attributed to its starch structure, specifically to the higher content of amylose compared to amylopectin. Amylose is digested more slowly than amylopectin, leading to more efficient reduction in blood glucose and insulin levels after meals and prolonged satiety [2].

Additionally, the digestibility and biological value of einkorn can be enhanced through fermentation. Fermentation, one of the oldest biotechnological approaches used in food preservation, typically involves the chemical conversion of carbohydrates (e.g., sugars or starches) into alcohols and organic acids by pro-technological bacteria, such as yeasts and lactic acid bacteria [9].

A previous study comparing einkorn and wheat breads found that einkorn breads fermented with lactic acid bacteria and yeast significantly reduced the gastrointestinal response of glucose-dependent insulinotropic polypeptide (GIP), a hormone that plays a crucial role in regulating insulin secretion [10].

Among cereal-based fermented food products, non-alcoholic beverages are receiving increasing attention, driven by lifestyle changes that reflect a broader trend toward health-conscious nutrition [11,12]. These products, rooted in the traditions of developing countries (e.g., Boza, Bushera, Kvass, Togwa), represent cheaper alternatives to dairy probiotic beverages [13,14]. Moreover, novel cereal-based beverages have emerged in recent decades, using different grains and selecting appropriate starter cultures able to accelerate and standardize fermentation processes [15,16]. Indeed, the selection of lactic acid bacteria based on the cereal type is crucial to ensure properties such as improved micronutrient bioavailability [17], enhanced sensory properties, and extended shelf life [18].

Given these premises, this study aimed to evaluate the feasibility of producing an innovative einkorn-based non-alcoholic fermented beverage. To this end, twenty lactic acid bacteria cultures, originally isolated from commercial Boza (a traditional Bulgarian cereal-based beverage), were first evaluated as monocultures in mini-batch fermentation assays to assess their acidifying activity in a specifically formulated einkorn-based substrate. Subsequently, a starter culture comprising the strains that demonstrated the highest performance was formulated and further utilized to manufacture laboratory-scale prototypes of non-alcoholic fermented beverages. The prototypes were analyzed immediately after production and throughout their shelf life at +4 °C to assess their physico-chemical (pH, titratable acidity), microbiological (viable counts of presumptive mesophilic lactic acid bacteria, mesophilic aerobic bacteria, spore-forming bacteria, yeasts, and Enterobacteriaceae), chemical (total phenolic content, antioxidant activity, organic acids, sugars, and volatile profile), and gross compositional/nutritional (moisture, dry matter, protein, lipids, nitrogen-free extract, total dietary fiber, and ash) characteristics. Additionally, a sensory evaluation of the prototype was conducted before and after fermentation.

## 2. Materials and Methods

### 2.1. Selection of Cereal-Sourced Lactic Acid Bacteria

Twenty lactic acid bacteria cultures (Table 1) were preliminary assayed in mini-batch fermentations for their ability to ferment a substrate made with einkorn wheat. All lactic acid bacteria cultures had previously been isolated from commercial samples of Bulgarian Boza [19] and subjected to molecular typing by a Randomly Amplified Polymorphic DNA (RAPD) analysis. They were stored as frozen cultures in a mixture of glycerol and Man, Rogosa, and Sharp (MRS) broth (VWR International, Milan, Italy), at a 2:3 (*v v*^−1^) ratio. Prior to use, each strain was propagated on MRS agar (VWR) incubated at 30 °C for 48 h for mini-batch fermentations, or sub-cultured in MRS broth (VWR) incubated at 30 °C for 24 h, for the manufacturing of the laboratory-scale prototypes.

### 2.2. Einkorn Wheat Purchase

In total, 2.4 kg of einkorn wheat grains was purchased from a local retailer (Naturasì, Ancona, Italy). The nutritional composition of the grains per 100 g was as follows: carbohydrates, 63.8 g; proteins, 11.9 g; lipids, 3 g; fibers, 7.7 g; salt, 0 g; and a caloric value of 346 Kcal.

### 2.3. Preparation of Einkorn Wheat-Based Substrate for Mini-Batch Fermentations

**Figure 1:**
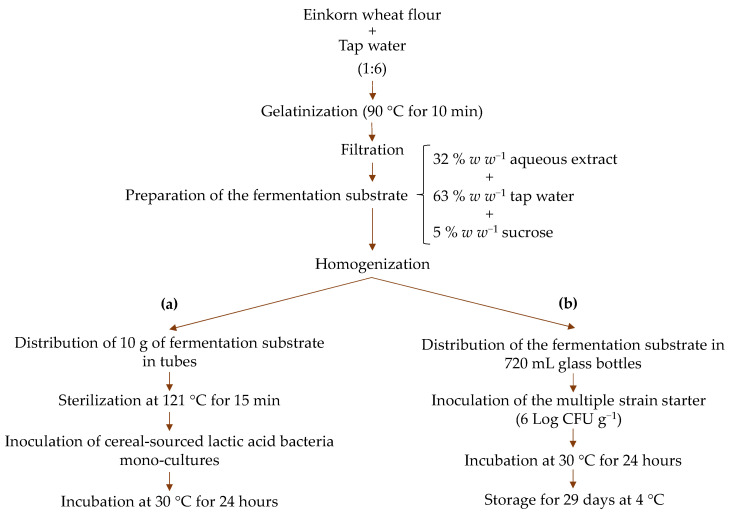
A flow chart detailing the steps for preparing the fermentation substrate to be exploited in the mini-batch fermentations (**a**) and laboratory-scale prototypes (**b**).

Figure 1a reports the flow chart for the preparation of the fermentation substrate exploited in the mini-batch fermentations.

Briefly, approximately 100 g of einkorn wheat was coarsely milled with a manual grinder until a granulate with a diameter of ~1.5 mm was obtained. The granulate was then added with 600 mL of tap water and heated to 90 °C for 10 min to induce gelatinization (stirring gently during the process). The resulting mixture was filtered using a fine mesh strainer. Subsequently, the obtained filtrate (approx. 300 g) was diluted with tap water and added with sucrose at a ratio of 32% filtrate (*w w*^−1^), 63% tap water (*w w*^−1^), and 5% sucrose (*w w*^−1^). Ten grams of aliquots of the resulting substrate was distributed in 50 mL tubes and finally sterilized at 121 °C for 15 min.

### 2.4. Mini-Batch Fermentations for the Selection of a Multiple-Strain Starter for the Manufacturing of Laboratory-Scale Prototypes

In total, 20 strains of lactic acid bacteria (Table 1) were separately inoculated, in triplicate, into the substrate prepared as described in Section 2.3. For each strain, an aliquot of the bacterial biomass harvested from the MRS plates was inoculated with a sterile disposable loop (1 μL) under sterile conditions to an initial cell density of ~7 Log CFU g^−1^. The substrates were then anaerobically incubated at 30 °C for 24 h. Aliquots (1 g) were collected under sterile conditions immediately after inoculation (*t*_0_), as well as after 8 (*t*_1_) and 24 (*t*_2_) hours of incubation, and subjected to pH measurement. The pH values were measured using a pH meter (Benchtop pH meter, pH50, VioLab Basic, XS Instruments, Carpi, Italy); the results were expressed as mean pH values ± the standard deviation of three replicates.

The three strains characterized by the best fermentation performance were selected based on their acidifying activity. This assessment included evaluating both the acidification rate (pH < 5.5 after 8 h fermentation) and acidification extent (pH < 4.8 after 24 h fermentation) [15]. The selected strains were included in the formulation of a multiple-strain starter to be exploited to produce laboratory-scale prototypes.

### 2.5. Production of Laboratory-Scale Prototypes

The laboratory-scale prototypes of yogurt-like einkorn-based fermented beverages were manufactured as described in Figure 1b. Briefly, ~2 kg of einkorn wheat grains was processed as previously described in Section 2.3. Aliquots (500 g) of the homogenized substrate were distributed into 720 mL glass bottles and inoculated, in triplicate, with the selected multiple-strain starter at a final concentration of ~6 Log CFU g^−1^ (started beverage, Sb). Additionally, a non-inoculated control (control beverage, Cb) was also prepared, in triplicate.

The inoculum, containing the strains BZ33, BZ34, and BZ47 in a 1:1:1 ratio, was prepared by sub-culturing each lactic acid bacteria strain in MRS broth (VWR) incubated at 30 °C for 24 h; after growth, the medium was removed by centrifugation at 4000 rpm for 10 min, and the cell pellets were washed with a sterile 0.85% (*w v*^−1^) NaCl saline solution, and finally resuspended in sterile water. The cell concentration of the inoculum was determined by optical reading at 600 nm (OD600 = 1 = 8.70 Log CFU mL^−1^) using a UV–Vis Shimadzu UV-1800 spectrophotometer (Shimadzu Corporation, Kyoto, Japan). All the prototypes were subsequently incubated at 30 °C for 24 h and then stored at 4 °C for 29 days.

### 2.6. Chemicals

Sodium carbonate, gallic acid, Trolox (6-hydroxy-2,5,7,8- tetramethylchroman-2-carboxylic acid), Ferric-Reducing Antioxidant Powder (FRAP), hydrochloric acid, ferric chloride hexahydrate, 2, 4, 6-tripyridyl-s-triazine (TPTZ), and sodium acetate were purchased from Sigma-Aldrich (St. Louis, MO, USA). Folin–Ciocalteu’s phenol reagent solution was obtained from VWR (Milano, Italy). Analytical standards of organic acids, sugars, and polyalcohols (lactic acid, glutaric acid, malic acid, cinnamic acid, ferulic acid, gallic acid, glucuronic and galacturonic acid, caffeic acid, coumaric acid, glucose, fructose, trehalose, maltose, galactose, arabinose, rhamnose, fucose, ribose, xylose, mannose, melibiose, palatinose, trehalose, and sucrose) were all purchased from Sigma-Aldrich (St. Louis, MO, USA). Furthermore, for extraction and derivatization steps, di-methylformamide (DMF), trimethylchlorosilane (TMCS), hexamethyldisilane (HMDS), and n-hexane were also obtained from Sigma Aldrich (St. Louis, MO, USA). Toluene, used as an internal standard, and sodium chloride (NaCl) were obtained from Carlo Erba reagents (Milan, Italy) and Sigma-Aldrich (St. Louis, MO, USA), respectively. β-Phenyl glycoside and glutaric acid, used as internal standards, were purchased from Sigma-Aldrich (St. Louis, MO, USA).

### 2.7. Analysis of Prototypes

Started prototypes (Sb) and uninoculated controls (Cb) were sampled for physico-chemical, microbiological, and chemical analyses immediately after the inoculation of the starter (*t*_0_), after 24 h of fermentation (*t*_1_), and at regular intervals after 2, 3, 6, 8, 10, 13, 15, 17, 20, 22, 24, 27, and 29 days of storage at +4 °C. In more detail, they were subjected to pH measurements (Section 2.7.1) at all the sampling times; titratable acidity (TA) determination (Section 2.7.1) at *t*_0_, *t*_1_, and *t*_29_; and microbiological analyses (Section 2.7.2) at *t*_0_, *t*_1_, *t*_8_, *t*_15_, *t*_22_, and *t*_29_. Furthermore, total phenolic content determination (Section 2.7.3), antioxidant capacity evaluation (Section 2.7.3), volatile organic compound (Section 2.7.4), organic acid and sugar (Section 2.7.5) analyses were performed at *t*_0_, *t*_1_, and *t*_29_. The prototypes were also subjected to proximate composition determination (Section 2.7.6) after fermentation (*t*_1_) and to a sensory analysis (Section 2.7.7) at *t*_0_ and *t*_1_.

#### 2.7.1. Determination of pH and Titratable Acidity (TA)

The determination of pH was performed as previously described in Section 2.4. Titratable acidity (TA) was determined as detailed by Rampanti et al. [20]. Briefly, 10 g aliquots were homogenized with 90 mL of distilled water and titrated with a 0.1 N solution of NaOH to reach a fixed pH of 8.3. TA was expressed as % of lactic acid equivalents according to the following formula:

(1) $$TA=\frac{volume\;of\;titrant\times N\times 90}{weight\;of\;sample\times 1000}\times 100$$

where *N* is the concentration expressed as normality of the titrant, and 90 is the equivalent molecular weight of lactic acid. The results were expressed as mean values ± the standard deviation of three replicates.

#### 2.7.2. Microbiological Analyses

Aliquots (10 g) of each prototype were added with 90 mL of sterile 0.1% (*w v*^−1^) peptone water and homogenized using a Stomacher apparatus (400 Circulator, International PBI, Milan, Italy) for 2 min at 230 rpm. The resulting homogenates were serially ten-fold diluted in sterile 0.1% (*w v*^−1^) peptone water and subjected to the enumeration of (i) presumptive mesophilic lactic acid bacteria on MRS agar (VWR) supplemented with cycloheximide (VWR) (100 mg L^−1^) incubated at 30 °C for 48−72 h; (ii) mesophilic aerobic bacteria on Plate Count Agar (PCA) (VWR) incubated at 30 °C for 72 h; (iii) yeasts on Rose Bengal Chloramphenicol agar (VWR) incubated at 25 °C for 5 days; (iv) Enterobacteriaceae on Violet Red Bile Agar (VWR) incubated at 37 °C for 24 h; (v) spore-forming bacteria on PCA incubated at 30 °C for 72 h, after heat treatment of the homogenate at 80 °C for 10 min, followed by cooling in iced water. The results were expressed as the mean Log CFU g^−1^ of the beverage of three replicates ± standard deviation.

#### 2.7.3. Total Phenolic Content (TPC) Determination and Antioxidant Capacity (AOC) Evaluation

For the assessment of antioxidant compounds, 2 g of fresh samples was weighed and centrifuged (Centrifugette 4206 centrifuge, Alc International, Pévy, France) at 12,000 rpm for 10 min at room temperature. Subsequently, the concentration of polyphenols and antioxidant components was determined by applying the tests described below.

The total phenolic content (TPC) was determined using the Folin-Ciocalteau method based on the protocol of Martin- Diana et al. [21] with few modifications. Briefly, 250 µL of the sample supernatant was added to 1 mL of Folin–Ciocalteu’s reagent diluted in water (1/10) and 2 mL of a sodium carbonate aqueous solution (10% *w v*^−1^) and kept in the dark for 30 min at room temperature. A spectrophotometer (JASCO V-530 spectrophotometer, Easton, MD, USA) was used to determine the absorbance at 760 nm. A standard of gallic acid prepared in the range of 10–100 mg kg^−1^ (5 points) was analyzed in the same conditions as the samples to build a calibration curve, allowing the evaluation of the TPC of samples. All the analyses were performed twice, performing three consecutive absorbance measurements on each replicate, and the TPC results were expressed as mg of gallic acid equivalent on kg (mg GAE kg^−1^).

The antioxidant capacity of the prototypes was evaluated by a Ferric-Reducing Antioxidant Power (FRAP) assay as reported by Önder et al. [22] with few modifications. Briefly, the FRAP working solution was prepared by mixing 2.5 mL of an aqueous solution of ferric chloride hexahydrate (20 mM) with 2.5 mL of an aqueous solution of TPTZ (10 mM) acidified with hydrochloric acid (40 mM) in 25 mL of an acetate buffer (300 mM), pH 3.6. Before the analysis, the FRAP working solution was warmed at 37 °C for 30 min. In total, 150 µL of the beverage sample or blank was added to 2850 µL of the FRAP working solution and kept in the dark for 30 min at room temperature; then, the absorbance was recorded in triplicate at 593 nm (JASCO V-530 spectrophotometer, Easton, MD, USA). The ferric ion-reducing activity of samples was estimated based on Trolox, used as a reference, by building a calibration curve (100–1000 µM; 5 points). The analyses were conducted in duplicate, and the results were expressed as µM TEAC.

#### 2.7.4. Determination of Volatile Organic Compounds (VOCs) by HeadSpace Solid-Phase MicroExtraction (HS-SPME) Coupled to Gas Chromatography–Mass Spectrometry (GC-MS)

The determination of volatile organic compounds (VOCs) was conducted according to Dall’Asta et al. [23] with slight modifications. Briefly, 5 g of each prototype was placed in 20 mL SPME vials with 0.5 g of NaCl, and analyzed in triplicate on a Thermo Scientific Trace 1300 gas chromatograph coupled with a Thermo Scientific ISQ (Thermo Fisher, Waltham, MA, USA) single quadrupole mass spectrometer, equipped with an electronic impact ion source. In total, 5 µL of an aqueous solution of toluene (100 µg mL^−1^) was used as an internal standard, and added in each vial to allow a semi-quantification of volatile compounds.

The vials were placed into a thermostat oven of the autosampler (HT280T GC Autosampler, HTA Brescia, Italy) for 15 min to equilibrate the mixture. The fiber was then introduced in the sample head space, and kept there for 30 min. A silica fiber coated with 50/30 µm Divinylbenzene–Carboxen–Polymethylsiloxane (DVB/Carboxen/PDMS) (Supelco, Bellefonte, PA, USA) was employed for each Solid-Phase MicroExtraction (SPME) analysis. Upon the sampling time, the fiber was taken out of the vial, and inserted into the GC-MS injector where the volatiles were allowed to desorb for 2 min at 250 °C.

A ZB-WAX plus capillary column (Phenomenex, 30 m × 0.25 mm, ft × 0.25 mm) was used for separation. The injections were carried out in the splitless mode and helium was used as carrier gas. The column temperature was initially set at 50 °C for 3 min; and then it increased to 200 °C at 5 °C/min, kept for 12 min.

The full scan (from 41 to 500 m/z) MS acquisition mode was used, and the detector temperature was set at 230 °C. Peak identification was carried out by matching registered mass spectra with those found in the instrument library (NIST 14). Linear retention indices (LRIs) were calculated for each observed signal based on the retention time of a C_8_–C_20_ alkane standard solution (Sigma-Aldrich, St. Louis, MO, USA) tested under the same GC conditions used for the sample analysis. All the identified compounds were semi-quantified using toluene as a reference.

#### 2.7.5. Determination of Organic Acids and Sugars by GC-MS

Sugars, polyalcohols, and organic acids of prototypes were determined following the protocol described by Cirlini et al. [24] with slight modifications. For extraction, one gram of each sample was added with 10 mL of distilled water in a 15 mL Falcon tube and placed on a laboratory shaker (HS 501 digital shaker, IKA-Werke GmbH & Co, Staufen, Germany) set at 200 strokes/minute at room temperature for 10 min, and then centrifuged for 10 min at 5000 rpm (Centrifugette 4206 centrifuge, Alc International, Pévy, France). Subsequently, 1 mL of each extract was added to 1 mL of an aqueous internal standard solution (β-phenyl glycoside and glutaric acid, 500 ppm in 50 mL), and evaporated to dryness using a rotary evaporator set at 40 °C. The residue was then recovered with 0.5 mL of DMF, and added with 0.3 mL of TMCS and 0.6 mL of HMDS. The resulting mixtures were transferred into 4 mL vials and heated at 80 °C for 30 min to obtain analyte trimethyl-silyl-ethers. The identification of GC-MS signals was performed using reference standards of organic acids, sugars, and polyalcohols. Prior to the GC-MS analysis, the derivatized compounds were extracted at room temperature using 1 mL of n-hexane. A 1 µL aliquot of each derivatized sample was injected into a Thermo Scientific Trace 1300 gas chromatograph coupled to a Thermo Scientific ISQ mass spectrometer equipped with an electronic impact (EI) source (Thermo Fisher, Waltham, MA, USA). A DB-5MS capillary column (30 m × 0.25 mm, ft × 0.25 mm, Agilent Technologies, Santa Clara, CA, USA) was employed for separation using helium as carrier gas. The column temperature was initially set and maintained for 3 min at 60 °C; the oven temperature was increased by 10 °C/min until 280 °C. The final temperature was kept for 5 min, with a total run time of 30 min. The injection was performed in the split mode with a split ratio of 1/20. The range between 41 and 500 m/z was selected, and the full scan mode was used as the acquisition mode. The analysis was performed in triplicate. The identification of GC-MS signals was performed by evaluating both the results obtained from analyses of several sugar, polyalcohol, and organic acid standards subjected to the same sample preparation and treatment, and by comparing registered mass spectra with those available in the instrumental library (NIST14). The semi-quantification of selected gas-chromatographic signals was carried out using the internal standards: glutaric acid for the organic acid fraction and β-phenyl glycoside for sugars and polyalcohols, respectively.

#### 2.7.6. Proximate Composition and Energy Content Determination

The started and control prototypes were subjected to proximate composition determination as reported by Cardinali et al. [15]. Briefly, (i) moisture content and dry matter were assessed through the gravimetric method (AOAC Official Method 950.46), (ii) ash was evaluated in a convection oven (AOAC, 920.153), (iii) total dietary fiber was assessed through a gravimetric/enzymatic method (AOAC, 991.43), (iv) proteins were evaluated by the Kjeldahl method (AOAC, 981.10), (v) lipids were determined by Soxhlet extraction (AOAC, 991.36), and (vi) carbohydrates were calculated by subtracting the determined amount of ash, proteins, and lipids from the determined total dry matter. The results were expressed as mean g 100 g^−1^ beverage ± the standard deviation of three replicates.

The energy content was calculated considering the contribution of all the above determined macronutrients and was reported as Kcal 100 g^−1^ beverage ± the standard deviation of three replicates.

#### 2.7.7. Sensory Analysis

The sensory analysis was carried out according to Coda et al. [25] with slight modifications. A total of 50 not-trained panelists (male and female in equal distribution; between 19 and 50 years of age) were recruited to assess the flavor characteristics of the einkorn-based beverage, especially in terms of odor perception, before (Sb t_0_) and after lactic acid fermentation (Sb t_1_), respectively. Each attribute was scored on a scale of 0–3 (low), 4–6 (medium), and 7–10 (high); five sensory attributes were selected, including yogurt, cereal, toasted, fruity, and vegetable notes. In addition, overall acceptability was also evaluated on a similar score scale. Samples were served in glass vials (5 g in a 20 mL vial), previously labeled with random three-digit codes, and served at room temperature (20 °C). Mean scores for all attributes of the einkorn-based beverage before (Sb t_0_) and after lactic acid fermentation were used for graphical representation, including spider plots depicting different intensity levels.

### 2.8. Statistical Analysis

The results of physico-chemical analyses, microbial enumeration, and proximate composition determinations were subjected to a one-way analysis of variance (ANOVA) through the Tukey–Kramer honest significant difference (HSD) test (*p* ≤ 0.05), using JMP Version 11.0.0 software (SAS Institute Inc., Cary, NC, USA).

Data from chemical and sensory analyses were elaborated on by using SPSS ver.28 (SPSS Inc., Chicago, IL, USA). In detail, one-way ANOVA was used to compare the concentration of polyphenols, antioxidant compounds, volatile compounds, organic compounds, sugars, and polyalcohols of started and control samples, as well as score values obtained from sensory evaluations. Post hoc Tukey’s test was used to determine significant differences among samples (*p* ≤ 0.05). Furthermore, a Pearson correlation analysis was employed to evaluate the association between TPC and FRAP results. Moreover, a Principal Component Analysis and a heatmap were realized using Metaboanalyst 5.0 in order to evaluate differences and/or relationships between started and control samples. In more detail, data including concentrations of organic acids, volatile compounds, sugar, TPC, and antioxidant activity were used as variables to perform both statistical treatments. For PCA, all data were normalized using log transformation (base 10), while the hierarchical clustering heatmap was achieved using normalized data and feature autoscaling (distance method: Euclidean; clustering method: ward).

## 3. Results and Discussion

### 3.1. The Formulation of a Multiple-Strain Starter for the Manufacturing of Laboratory-Scale Prototypes Through Mini-Batch Fermentations

The einkorn-based substrate used for the mini-batch fermentations was characterized by an initial pH value of 7.37 ± 0.04. The results of the pH measurements carried out during the fermentation period are reported in Table 1. In detail, after 8 h of incubation, pH values generally remained above 5.5, ranging between 7.30 ± 0.14 (*Pediococcus parvulus* strain BZ39) and 5.55 ± 0.02 (*Lacticaseibacillus casei/paracasei* strain BZ47). However, after 24 h of fermentation, pH dropped below 4.8 in most samples, with values ranging between 5.91 ± 0.27 (*Pediococcus parvulus* strain BZ39) and 3.71 ± 0.01 (BZ33, *Lacticaseibacillus casei* strain BZ33). The three strains with the best acidification extent, namely BZ33 (pH_24h_: 3.71 ± 0.01), BZ34 (pH_24h_: 3.85 ± 0.03), BZ47 (pH_24h_: 3.78 ± 0.03), were selected for the formulation of the multiple-strain starter.

The selection of a starter culture exhibiting good technological properties (i.e., high acidification activity) for each specific cereal substrate is a crucial step for a fast and standardized fermentation, thus enabling the production of safe, nutrient-rich fermented foods with desirable flavor attributes [16,26].

### 3.2. pH and Titratable Acidity (TA) of Control and Started Prototypes

**Figure 2:**
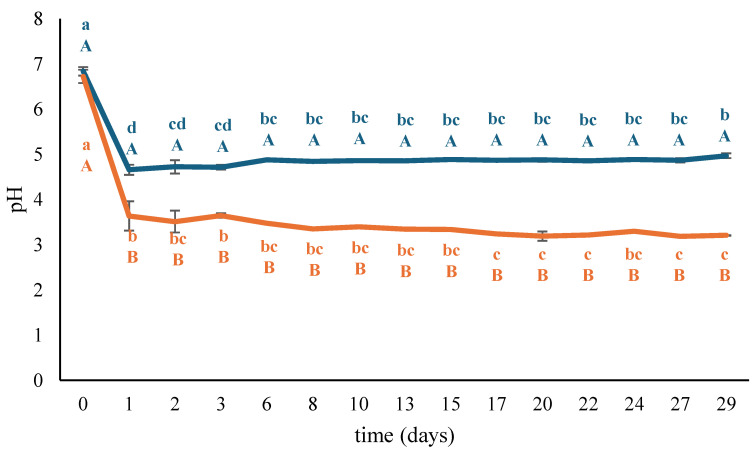
Results of pH measurements performed on started (Sb, orange line) and control (Cb, blue line) prototypes of non-alcoholic einkorn-based fermented beverages. The results are reported as mean values of three replicates ± standard deviations. For each prototype, values labeled with different lowercase letters in the line are significantly different (*p* ≤ 0.05), whereas for each sampling time, values labeled with different capital letters are significantly different (*p* ≤ 0.05) in started and control prototypes.

The results of pH measurements performed on control (Cb) and started (Sb) prototypes of fermented non-alcoholic einkorn-based beverages are depicted in Figure 2. Prior to the inoculation of the multiple-strain starter (t_0_), pH values were 6.84 ± 0.10 and 6.72 ± 0.15 in the control and started samples, respectively. After 24 h of fermentation (t_1_), a significant pH reduction was observed in all the prototypes, with significantly lower values in Cb_t1_ (4.66 ± 0.11) and Sb_t1_ (3.64 ± 0.32). Moreover, inoculation with the multi-strain starter resulted in significantly lower pH values in the inoculated samples compared to the controls, with the pH continuing to decrease significantly during storage at 4 °C, ultimately reaching a value of 3.21 ± 0.01 (Sb_t29_). Conversely, the pH of control samples increased during refrigerated storage, reaching a value of 4.97 ± 0.05 (Cb_t29_). Notably, after 24 h (t_1_), the pH value in the beverage inoculated with the multiple-strain starter closely corresponded to the pH levels observed in the single-strain mini-batch fermentations of the einkorn-based substrate inoculated with isolates BZ33, BZ34, and BZ47.

The lactic acid bacteria strains herein assayed showed good acidifying properties in the einkorn-based substrate under study, thus supporting the results previously reported by Cardinali et al. [15] for the same strains inoculated in red rice-, barley-, and buckwheat-based substrates. In addition to the well-documented ability of cereals to support lactic acid bacteria growth [15], the addition of sugar to the fermentation substrate as an immediately available carbon source may have further promoted bacterial growth and, consequently, increased acidification [27,28].

The pH values of the started laboratory-scale prototypes after fermentation are consistent with those reported by Salmerón et al. [29], who detected pH values ranging between 3.3 and 3.7 in fermented beverages based on oat, barley, and malt. In fermented beverages, the reduction in pH is crucial for microbiological stability; pH values below 3.8 generally inhibit pathogens and most food spoilage bacteria growth [29,30].

As for TA, both samples showed values of 0.03 ± 0.00% of lactic acid equivalents at t_0_. After 24 h of fermentation (t_1_), a significant increase in TA was observed in both prototypes, with significantly higher values in the started sample (Sb_t1_: 0.23 ± 0.04% of lactic acid equivalents) compared to the control one (Cb_t1_: 0.10 ± 0.00% of lactic acid equivalents). During refrigerated storage, TA significantly increased in the started samples, reaching a final value of 0.43 ± 0.03% of lactic acid equivalents, while remaining stable in the control samples.

In lactic acid-fermented foods, pH reduction is mainly related to lactic acid production through lactic acid bacteria fermentative metabolism [31]. Therefore, titratable acidity is commonly expressed as % of lactic acid equivalents. The results herein collected for TA align with the quantification of organic acids performed by the GC-MS analysis. In more detail, TA was overestimated at t_0_ in both prototypes because other organic acids (i.e., malic acid) contributed to the final titratable acidity. On the contrary, the results obtained at t_1_ and t_29_ agreed well with the quantification results.

### 3.3. Viable Microorganisms in Control and Started Prototypes

The results of viable counting performed on control (Cb) and started (Sb) prototypes of the fermented non-alcoholic einkorn-based beverages are reported in Table 2. At t_0_, presumptive mesophilic lactobacilli were below the detection limit (<1 Log CFU g^−1^) in control beverages, whereas they attested at a load of 6.3 ± 0.0 Log CFU g^−1^ in started beverages immediately after the inoculation. The incubation at 30 °C allowed this microbial group to grow in all the prototypes, reaching significantly higher load in the started samples (Sb_t1_: 9.0 ± 0.0 Log CFU g^−1^) compared to the control ones (Cb_t1_: 5.1 ± 0.4 Log CFU g^−1^). Presumptive mesophilic lactobacilli maintained their viability during the storage, with loads at t_29_ of 9.1 ± 0.0 Log CFU g^−1^ (Sb_t29_) and 4.5 ± 0.8 Log CFU g^−1^ (Cb_t29_).

As already suggested by pH and TA determinations, both the inoculated and endogenous lactic acid bacteria were able to grow in the einkorn-based beverage and to remain viable during storage. In the started prototypes, lactic acid bacteria growth during fermentation and their survival during storage agreed well with the results collected by Cardinali et al. [15] in red rice-, barley-, and buckwheat-based beverages. On the contrary, Coda et al. [25] described a reduction in the load of lactic acid bacteria during 30 days of refrigerated storage up to ~1 Log CFU g^−1^. Maintaining high LAB viability during storage is an important aspect to be considered in the development of plant-based beverage alternatives to milk-based beverages [32]. In the control samples, lactic acid bacteria enumeration resulted again in contrast with the findings of Coda et al. [25], who enumerated ~2 Log CFU g^−1^ lactic acid bacteria, with no significant changes during fermentation and storage. In the control prototypes of the present study, lactic acid bacteria naturally contaminating the einkorn substrate [33] might have survived the heat treatment applied during gelatinization, potentially as sublethal injured cells [34], and likely recovered and grew during incubation at 30 °C.

Mesophilic aerobic bacteria followed the same trend of lactic acid bacteria, with loads of 3.1 ± 0.4 Log CFU g^−1^ (Cb_t0_) and 6.3 ± 0.0 Log CFU g^−1^ (Sb_t0_) at t_0_ and increasing to 4.7 ± 0.7 Log CFU g^−1^ (Cb_t29_) and 9.1 ± 0.0 Log CFU g^−1^ (Sb_t29_) at the end of the storage.

Spore-forming bacteria attested at ~2 Log CFU g^−1^ in all the prototypes at t_0_. In the started samples, they remained stable during fermentation and storage, whereas in control samples, they increased to 4.5 ± 0.7 Log CFU g^−1^ (Cb_t1_) after fermentation and slightly decreased during the storage. However, no statistically significant differences in spore-forming bacteria loads were observed between started and control prototypes at each sampling time (Table 2).

Spore-forming bacteria may have been naturally present in raw materials as spore-form contaminants [35] and survived the applied heat treatment during gelatinization [36]. Their outgrowth would only occur under suitable conditions [37]. Despite this microbial group including both pathogenic and spoilage bacteria [35], most of them are unable to grow at the low pH (Sb_t1_: ~3.6; Sb_t29_: ~3.2) observed in the started prototypes [37]. Conversely, the pH values recorded in control samples (Cb_t1_: ~4.7; Cbt_29_: ~5.0) might support the growth of the pathogen *Clostridium botulinum* [37].

Yeasts and Enterobacteriaceae were below the detection limit (<1 Log CFU g^−1^) in both started and control samples at all sampling times. They were likely eliminated during the heat treatment applied with the gelatinization step [36].

### 3.4. Total Phenolic Content and Antioxidant Activity of Control and Started Prototypes

Total phenolic content and antioxidant activity of control (Cb) and started (Sb) prototypes of fermented non-alcoholic einkorn-based beverages are reported in Table 3. In more detail, concerning TPC, the started einkorn-based beverage showed a significant increase from 13.00 ± 1.41 mg GAE kg^−1^ at t_0_ to 24.29 ± 5.69 mg GAE kg^−1^ at t_1_ and 32.37 ± 6.71 mg GAE kg^−1^ at t_29_. The same trend was also observed for control samples. No statistically significant differences were observed between started and control prototypes at t_29_.

The results obtained for fermented einkorn-based beverages in this study were lower than those found by Coda et al. [38] in non-alcoholic emmer fermented beverages stored for 30 days, which were made with different percentages of gelatinized emmer flour. In detail, the authors found TCP contents of 52.73 mg GAE kg^−1^ and 154 mg GAE kg^−1^ in beverages containing 10% and 30% (*w w*^−1^) of gelatinized emmer flour, respectively [38]. Amarowicz et al. [39] suggested that the diminished TPC in Kvass, a popular cereal-based fermented beverage in Central and Eastern Europe, could be related to the filtration step in the production process, which removes cereal particles from the beverages.

As for AOC, no statistically significant differences were observed between Cb (100.31 ± 18.02 μM) and Sb (83.58 ± 14.82 μM) at t_1_. Conversely, the AOC of the started prototype was significantly lower compared to the control one at t_29_ (166.83 ± 22.63 vs. 88.67 ± 6.32 μM in Cb_t29_ and Sb_t29_, respectively). Concerning the antioxidant activity, Amarowicz et al. [39] reported values of ABTS ranging from 133 to 1000 μM in Kvass. Thus, the variability in the content of phenolic compounds and antioxidant activity in cereal-based fermented beverages could be attributed to the different raw materials and conditions of the production process.

The Pearson correlation coefficient revealed a direct relation between TPC and FRAP values, resulting in a value range of 0.51, indicating a strong positive correlation between the amount of polyphenols and the antioxidant activity [40].

### 3.5. VOC Profile of Control and Started Prototypes

A total of 41 compounds were detected in the fermented non-alcoholic einkorn-based beverages (Table 4). These compounds belonged to several chemical classes, including acids (7), alcohols (15), aldehydes (4), ketones (7), furans (2), esters (2), and miscellaneous compounds (4) (Table 5). The results of the semi-quantification of the identified volatile compounds are also reported in Table 5.

The volatile composition of the prototypes was affected by the storage time and the multiple-strain inoculum. For instance, acids were the predominant class identified in the started prototype. Acids increased in both started and control beverages during fermentation and storage, reaching a final concentration of 21.3 ± 3.4 and 4.7 ± 1.3 ng g^−1^, respectively, after 29 days. In more detail, started prototypes showed significantly higher concentrations of 2-ethyl butanoic acid, acetic acid, isobutyric acid, and caproic acid compared to control samples. The highest concentration of acetic acid was found in Sb_t29_, attesting at 13.1 ± 1.8 ng g^−1^.

The fermentation process exerted a significant influence on the volatile profile of the einkorn-based beverages, leading to the production of specific compounds such as the identified acids, which contributed to their acidic, cheesy, and fatty flavor notes. The increase in acetic acid during storage may be related to the activation of the acetate kinase route of the phosphogluconate pathway by lactic acid bacteria [41,42]. Regarding the volatile short-chain fatty acids in the fermented beverages (butyric acid, isobutyric acid, isovaleric acid, and caproic acid), their increase could arise from pyruvate fermentation through the glycolytic pathway or through the phosphoketolase pathway under heterofermentative conditions [43,44]. Furthermore, according to Blandino et al. [45], the acids identified in the einkorn wheat beverage are typical of fermented cereal-based foods.

In addition to acids, ketones were also significant compounds in the started prototypes and increased during storage, reaching a final total concentration of 18.6 ± 1.8 ng g^−1^. The main ketones detected after 29 days of storage were 2-heptanone, acetoin, and 2-nonanone (2.4 ± 0.6, 9.5 ± 0.6, and 4.5 ± 0.8 ng g^−1^, respectively). Their mean content was statistically higher in started samples compared to the control ones (*p* ≤ 0.05). Moreover, a few ketones, such as 2-undecanone and 4-acetonylcycloheptanone, were exclusively detected in the started prototypes.

Ketones are noted for imparting delightful odors to food, even at low concentrations. Most ketones are produced through the microbial oxidation of fatty acids or by decarboxylation pathways [46,47]. The main identified ketones provide cheesy, buttery, and fruity notes [48]. Similar findings were reported by Wang et al. [49] and Hadj Saadoun et al. [50], who found that lactic acid bacteria fermentation significantly increased ketone synthesis, including 2-heptanone and 2-nonanone, in kiwifruit juice and okara, respectively.

**Table 4:** Volatile compounds identified in non-alcoholic einkorn-based fermented beverages.

Peak N°	RT	Identification	Odor, Flavor Type ^a^	LRI ^b^	LRI ^c^	Reference
1	2.96	Ethanol	alcoholic	922	922	[51]
2	5.16	Hexanal	green	1106	1122	[52]
3	6.15	2-n-Butylfuran	spicy	1152	1140	[53]
4	6.22	o-Xylene	geranium/fruity	1156	1159	[52]
5	7.02	1-Butanol	fermented	1193	1165	[54]
6	7.5	2-Heptanone	cheesy	1214	1198	[55]
7	8.16	4-Methyl-2-heptanone		1240	1224	[56]
8	8.74	2-Pentylfuran	fruity	1263	1258	[57]
9	9.08	τ-Terpinene	terpenic	1277	1265	[58]
10	9.39	Isobutenylcarbinol	fruity	1290	1277	[55]
11	9.46	Pentanol	fermented	1292	1280	[55]
12	10.15	2-Octanone	earthy	1320	1304	[59]
13	10.26	Acetoin	buttery	1324	1312	[60]
14	10.74	Unknown				
15	11.21	2-Heptenal	green	1362	1334	[61]
16	12.17	Hexanol	herbal	1400	1392	[62]
17	12.62	Butanoic acid, 2-ethyl-	acidic	1419	MS	
18	12.93	2-Nonanone	fruity	1432	MS	
19	13.01	Nonanal	aldehydic	1435	1421	[63]
20	13.41	(+)-Santolina alcohol		1451	MS	
21	13.92	Benzene, 1,3-bis(1,1-dimethylethyl)-		1472	MS	
22	14.6	Acetic acid	acidic	1500	1460	[64]
23	14.64	1-Octen-3-ol	earthy	1502	1476	[65]
24	14.8	1-Heptanol	green	1509	1470	[57]
25	15.64	2-Ethyl-1-hexanol	citrus	1546	1518	[66]
26	16.22	Benzaldehyde	fruity	1571	1555	[55]
27	16.41	2-Nonanol	waxy	1579	1537	[67]
28	16.91	2,3-Butanediol	creamy	1601	1583	[68]
29	17.32	Octanol	waxy	1616	1585	[69]
30	17.5	Isobutyric acid	acidic	1623	1608	[70]
31	18.17	2-Undecanone	fruity	1647	1615	[67]
32	18.62	Benzoic acid methyl ester	phenolic	1664	1638	[71]
33	18.91	Butyric acid	cheesy	1675	1663	[72]
34	19.23	4-Acetonylcycloheptanone		1686	MS	
35	19.72	1-Nonanol	floral	1708	1673	[73]
36	19.87	Isovaleric acid	cheesy	1718	1706	[74]
37	22.25	2,5-Hexanediol, 2,5-dimethyl-		1857	MS	
38	23.71	Caproic acid	fatty	1931	1884	[74]
39	24.17	Propanoic acid, 2-methyl-3-hydroxy-2,2,4-trimethylpentyl ester		1955	MS	
40	25.05	Phenylethyl alcohol	floral	2002	1948 + MS	[75]
41	30.03	Nonanoic acid	waxy	2264	2181 + MS	[76]

^a^ Odor flavor type extracted from TheGoodScents Company. ^b^ Linear retention index calculated from C_8_ to C_20_ n-linear alkanes with ZB-wax capillary column. ^c^ Linear retention index reported in literature for ZB-wax capillary column or equivalent.

**Table 5:** Semi-quantification of volatile compounds of control (Cb) and started (Sb) non-alcoholic einkorn-based fermented beverages at different sampling times. Results are expressed as ng g^−1^.

Nº	Compound	Cb_t0_	Cb_t1_	Cb_t29_	Sb_t0_	Sb_t1_	Sb_t29_
Acids							
1	Butanoic acid, 2-ethyl-	n.d.	n.d.	n.d.	n.d.	1.59 ± 0.42 ^a^	1.36 ± 0.30 ^a^
2	Acetic acid	n.d.	0.89 ± 0.29 ^c^	1.13 ± 0.45 ^c^	n.d.	7.57 ± 0.89 ^b^	13.09 ± 1.76 ^a^
3	Isobutyric acid	n.d.	n.d.	n.d.	n.d.	0.27 ± 0.02 ^a^	0.24 ± 0.05 ^a^
4	Butyric acid	n.d.	0.27 ± 0.05 ^a^	0.30 ± 0.10 ^a^	n.d.	0.26 ± 0.07 ^a^	0.31 ± 0.13 ^a^
5	Isovaleric acid	n.d.	1.04 ± 0.17 ^b^	1.28 ± 0.40 ^a^	0.01 ± 0.01 ^c^	1.25 ± 0.36 ^a^	1.56 ± 0.49 ^a^
6	Caproic acid	n.d.	1.28 ± 0.15 ^c^	1.70 ± 0.35 ^c^	n.d.	3.22 ± 0.39 ^b^	4.38 ± 1.55 ^a^
7	Nonanoic acid	n.d.	0.23 ± 0.05 ^b^	0.25 ± 0.06 ^b^	n.d.	0.53 ± 0.05 ^a^	0.35 ± 0.10 ^b^
	Total	n.d.	3.72 ± 0.46 ^c^	4.66 ± 1.31 ^c^	0.01 ± 0.01 ^c^	14.69 ± 1.53 ^b^	21.28 ± 3.37 ^a^
Alcohols							
8	Ethanol	n.d.	2.15 ± 0.26 ^a^	2.76 ± 1.31 ^a^	n.d.	2.20 ± 0.23 ^a^	1.88 ± 0.34 ^a^
9	1-Butanol	n.d.	0.88 ± 0.21 ^a^	1.09 ± 0.18 ^a^	0.52 ± 0.03 ^b^	0.74 ± 0.08 ^a^	0.59 ± 0.10 ^ab^
10	Isobutenylcarbinol	0.07 ± 0.03 ^c^	0.08 ± 0.01 ^c^	0.10 ± 0.05 ^bc^	0.12 ± 0.05 ^b^	0.17 ± 0.04 ^b^	0.35 ± 0.09 ^a^
11	Pentanol	0.13 ± 0.01 ^c^	0.34 ± 0.01 ^b^	0.66 ± 0.35 ^a^	0.17 ± 0.05 ^c^	0.26 ± 0.05 ^b^	0.26 ± 0.06 ^b^
12	Hexanol	0.91 ± 0.31 ^b^	2.97 ± 0.61 ^a^	3.73 ± 3.08 ^a^	1.21 ± 0.25 ^b^	1.66 ± 0.22 ^b^	1.52 ± 0.14 ^b^
13	(+)-Santolina alcohol	0.53 ± 0.13 ^b^	0.74 ± 0.16 ^a^	1.04 ± 0.56 ^a^	0.67 ± 0.16 ^a^	0.84 ± 0.15 ^a^	0.79 ± 0.08 ^a^
14	1-Octen-3-ol	0.34 ± 0.07 ^b^	0.85 ± 0.26 ^a^	1.29 ± 0.50 ^a^	0.33 ± 0.04 ^b^	n.d.	n.d.
15	Heptanol	n.d.	0.74 ± 0.14 ^a^	1.00 ± 0.74 ^a^	n.d.	n.d.	n.d.
16	2-Ethyl-1-hexanol	0.10 ± 0.04 ^b^	0.28 ± 0.07 ^b^	0.61 ± 0.11 ^a^	0.09 ± 0.00 ^b^	0.15 ± 0.03 ^b^	0.55 ± 0.05 ^a^
17	2-Nonanol	n.d.	n.d.	n.d.	n.d.	n.d.	1.12 ± 0.14
18	2,3-Butanediol	n.d.	5.73 ± 1.80 ^a^	6.37 ± 3.70 ^a^	n.d.	1.45 ± 0.77 ^b^	1.39 ± 0.84 ^b^
19	Octanol	0.11 ± 0.01 ^b^	2.25 ± 0.96 ^a^	2.55 ± 1.21 ^a^	0.13 ± 0.02 ^b^	1.28 ± 1.06 ^a^	1.32 ± 1.17 ^a^
20	1-Nonanol	n.d.	1.24 ± 0.23 ^a^	1.63 ± 0.84 ^a^	n.d.	0.44 ± 0.06 ^c^	0.81 ± 0.11 ^b^
21	2,5-Hexanediol, 2,5-dimethyl-	0.48 ± 0.09 ^b^	0.24 ± 0.10 ^c^	0.37 ± 0.13 ^c^	0.64 ± 0.04 ^a^	0.24 ± 0.01 ^c^	n.d.
22	Phenylethyl alcohol	0.47 ± 0.04 ^a^	0.32 ± 0.05 ^b^	0.29 ± 0.16 ^b^	0.29 ± 0.04 ^b^	0.40 ± 0.01 ^ab^	0.26 ± 0.06 ^b^
	Total	3.14 ± 0.20 ^c^	18.81 ± 2.26 ^a^	18.03 ± 2.95 ^a^	4.17 ± 0.48 ^c^	9.83 ± 1.82 ^b^	10.85 ± 2.45 ^b^
Aldehydes							
23	Hexanal	n.d.	n.d.	0.97 ± 0.52 ^a^	n.d.	n.d.	0.37 ± 0.19 ^b^
24	2-Heptenal	n.d.	n.d.	n.d.	n.d.	n.d.	0.71 ± 0.38
25	Nonanal	0.09 ± 0.03 ^a^	0.11 ± 0.07 ^a^	n.d.	0.05 ± 0.00 ^a^	n.d.	n.d.
26	Benzaldehyde	n.d.	n.d.	0.17 ± 0.06 ^a^	n.d.	n.d.	0.09 ± 0.03 ^a^
	Total	0.09 ± 0.03 ^bc^	0.11 ± 0.07 ^bc^	0.84 ± 0.33 ^ab^	0.05 ± 0.00 ^c^	n.d.	1.21 ± 0.53 ^a^
Ketones							
27	2-Heptanone	n.d.	n.d.	n.d.	n.d.	1.46 ± 0.21 ^b^	2.42 ± 0.57 ^a^
28	4-Methyl-2-heptanone	0.81 ± 0.06 ^ab^	0.61 ± 0.05 ^b^	0.45 ± 0.10 ^b^	1.12 ± 0.30 ^a^	0.86 ± 0.07 ^a^	0.38 ± 0.08 ^b^
29	2-Octanone	n.d.	n.d.	n.d.	0.06 ± 0.02	n.d.	n.d.
30	Acetoin	n.d.	4.63 ± 1.08 ^c^	4.80 ± 1.34 ^c^	n.d.	6.82 ± 0.10 ^b^	9.53 ± 0.64 ^a^
31	2-Nonanone	n.d.	n.d.	0.36 ± 0.17 ^c^	n.d.	5.57 ± 0.59 ^a^	4.52 ± 0.76 ^b^
32	2-Undecanone	n.d.	n.d.	n.d.	n.d.	1.05 ± 0.22 ^a^	0.82 ± 0.09 ^a^
33	4-Acetonylcycloheptanone	n.d.	n.d.	n.d.	n.d.	1.61 ± 0.19 ^a^	0.96 ± 0.05 ^b^
	Total	0.81 ± 0.06 ^c^	5.24 ± 1.10 ^b^	4.64 ± 0.14 ^b^	1.18 ± 0.32 ^c^	17.37 ± 0.92 ^a^	18.64 ± 1.75 ^a^
Furans							
34	2-n-Butylfuran	n.d.	n.d.	n.d.	n.d.	n.d.	0.20 ± 0.11
35	2-Pentylfuran	0.09 ± 0.05 ^b^	0.34 ± 0.06 ^b^	0.30 ± 0.05 ^b^	0.19 ± 0.06 ^b^	0.65 ± 0.12 ^b^	1.81 ± 1.55 ^a^
	Total	0.09 ± 0.05 ^b^	0.34 ± 0.06 ^ab^	0.30 ± 0.05 ^ab^	0.19 ± 0.06 ^ab^	0.65 ± 0.12 ^ab^	2.01 ± 1.66 ^a^
Esters							
36	Benzoic acid, methyl ester	0.51 ± 0.04 ^a^	0.47 ± 0.03 ^b^	0.55 ± 0.35 ^b^	0.67 ± 0.18 ^a^	0.60 ± 0.05 ^a^	0.53 ± 0.07 ^a^
37	Propanoic acid, 2-methyl-, 3-hydroxy-2,2,4- trimethylpentyl ester	0.15 ± 0.03 ^a^	0.10 ± 0.02 ^a^	0.13 ± 0.08 ^a^	0.15 ± 0.05 ^a^	0.10 ± 0.03 ^a^	0.08 ± 0.02 ^a^
	Total	0.66 ± 0.04 ^ab^	0.57 ± 0.01 ^b^	0.49 ± 0.11 ^b^	0.82 ± 0.13 ^a^	0.70 ± 0.04 ^ab^	0.61 ± 0.09 ^ab^
Miscellaneous							
38	o-Xylene	0.14 ± 0.06 ^a^	0.21 ± 0.02 ^a^	0.16 ± 0.05 ^a^	0.15 ± 0.05 ^a^	0.20 ± 0.06 ^a^	n.d.
39	τ-Terpinene	0.30 ± 0.20	n.d.	n.d.	n.d.	n.d.	n.d.
40	Unknown	0.55 ± 0.11 ^a^	0.37 ± 0.03 ^a^	0.80 ± 0.61 ^a^	0.63 ± 0.25 ^a^	n.d.	n.d.
41	Ditert-butyl benzene	0.47 ± 0.22 ^b^	0.62 ± 0.05 ^b^	0.94 ± 0.32 ^ab^	1.07 ± 0.47 ^ab^	0.92 ± 0.05 ^ab^	1.24 ± 0.42 ^a^
	Total	1.37 ± 0.61 ^a^	1.57 ± 0.20 ^a^	1.61 ± 0.64 ^a^	2.23 ± 0.73 ^a^	1.12 ± 0.11 ^a^	1.24 ± 0.42 ^a^

Different letters in the same row indicate statistically significant differences among samples (*p* ≤ 0.05). n.d.: not detected.

A wide variability in compounds belonging to the alcohols’ group was observed in the fermented non-alcoholic einkorn-based beverages. Alcohols’ concentrations increased during storage in both started and control prototypes. However, this group represented the predominant class of volatiles in the control prototypes, with the highest concentrations in Cb_t1_ and Cb_t29_ ranging from 18.0 to 18.8 ng g^−1^, whereas they represented the third predominant class in the started prototypes, with concentrations ranging from 9.8 to 10.8 ng g^−1^ during storage. The main identified alcohols were hexanol, 2,3-butanediol, octanol, and nonanol. The alcohols identified in the einkorn-based beverages were correlated with fermented, herbal, green, and waxy flavor notes. 1-Butanol, 1-pentanol, 1-hexanol, and 2-ethyl-1-hexanol were previously identified in wheat-made fermented beverages by Coda et al. [38].

Other molecules, including aldehydes, furans, esters, and miscellaneous compounds, were also identified; however, their prevalence was lower compared to other chemical classes. Only one aldehyde, hexanal, was detected in both control and started prototypes after 29 days of storage. This compound provides a green flavor note and is considered a marker of lipid oxidation [77].

### 3.6. Characterization of Carbohydrates, Organic Acid Fractions, and Polyalcohols of Control and Started Prototypes

The glucidic and organic acid fractions of both started and control beverages were examined by the GC-MS analysis; 20 different signals were observed (Table 6). Two primary organic acids, specifically, lactic and malic acids, were identified in both prototypes. Lactic acid concentration significantly (*p* ≤ 0.05) increased in started samples during fermentation and storage (Table 6). As shown in Table 6, malic acid concentration was significantly reduced through fermentation, with values below the detection limit in all the prototypes at both t_1_ and t_29_.

Lactic acid, the most characteristic organic acid produced during fermentation by lactic acid bacteria (LAB), exhibited a notable increase in control beverages, likely attributed to the presence of endogenous microflora, as supported by the results of viable counting of presumptive mesophilic lactobacilli. The data herein collected for lactic acid in fermented einkorn-based beverages are in accordance with those previously reported by Kocková et al. [78] in a fermented pseudocereal substrate (amaranth flour). In detail, fermentation increased the amount of lactic acid from 402.90 mg kg^−1^ to 2217.50 mg kg^−1^ during storage [78]. Similarly, Coda et al. [25] observed a significant increase in lactic acid production (2538 mg kg^−1^ and 4032 mg kg^−1^, after 8 and 30 h, respectively) in a beverage made of a mixture of cereals, including rice and emmer. According to Muyanja et al. [79], lactate was one of the primary organic acids produced during the fermentation of Bushera, a popular traditional fermented product in Uganda, mainly made from sorghum flour. Again, the concentration of lactic acid increased during fermentation, specifically varying between 3400 mg kg^−1^ and 9600 mg kg^−1^ at 96 h. The high lactic acid content contributes to the improved preservation of fermented beverages due to the lower pH [30]. Several fermented products, including cereals, have preservation properties due to LAB metabolism [30]. By producing acid, LAB can lower the pH below 4, preventing the growth of pathogenic microorganisms, which can cause food spoilage, food poisoning, and diseases. As widely reported in the literature, lactic acid can also be produced by malolactic fermentation [80], through the metabolism of malic acid by decarboxylation to lactate [81]. The results of organic acids in this study showed how LAB metabolism, either from the inoculated starter or endogenous microflora, might have converted malic acid to lactic acid, resulting in a significant reduction in the former. The data collected overall highlighted that malic acid was converted into lactic acid during fermentation and storage, leading to a significant increase in the started samples both after 24 h (t_1_) and 29 days (t_29_).

Several polyalcohols and mono- and disaccharides have also been identified and quantified (Table 6). No statistically significant differences (*p* ≤ 0.05) in total polyalcohols’ profile were observed among the started beverage samples; however, a decrease in mannitol was observed in Sb_t1_ and Sbt_29_, respectively.

Glucose and fructose were the most abundant monosaccharides in all the analyzed samples. At t_0_, the started prototypes contained approximately 400 mg kg^−1^ of glucose and 120 mg kg^−1^ of fructose, respectively. After fermentation, a statistically significant increase (*p* ≤ 0.05) was observed in started prototypes (Table 6). Disaccharides in the fermented non-alcoholic einkorn-based beverages ranged between 18,975 and 20,340 mg kg^−1^.

The high level of monosaccharides in cereal-based fermented non-alcoholic beverages might derive from both residual added sucrose and the gelatinization step [25,82]. However, the data collected for fermented einkorn-based prototypes in this study are in contrast with those reported by Coda et al. [25], which showed a decrease in glucose (from 13,383 mg kg^−1^ to 12,356 mg kg^−1^) and fructose (from 14.418 mg kg^−1^ to 13,410 mg kg^−1^) from 8 h to 29 days in a beverage made with rice and emmer. The high concentration of disaccharides detected in the fermented samples might be tentatively ascribed to the hydrolysis of einkorn starch. This hypothesis is confirmed by Ziarno et al. [32], who proposed that starch hydrolysis can lead to the release of carbohydrates during the lactic acid fermentation of cereal and pseudocereal beverages. The amount and types of carbohydrates produced are influenced by various factors, including the type of cereal or pseudocereal used, the water content, the thermal treatment applied before fermentation, the bacteria involved in fermentation, and the process parameters. Our data seem to support the hypothesis of a partial starch hydrolysis during fermentation.

### 3.7. Principal Component Analysis (PCA) and Heatmap

**Figure 3:**
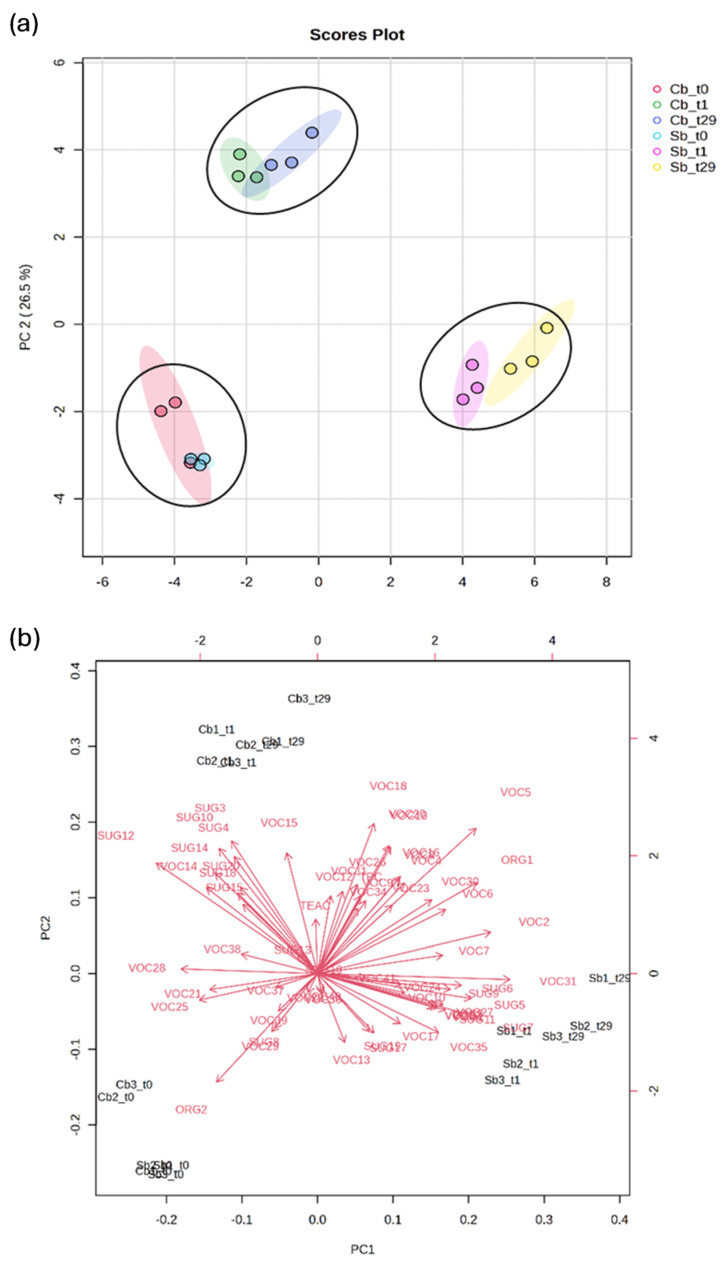
Principal Component Analysis (PCA) score plot (**a**) and relative loadings of variables (**b**) based on organic acids, volatile compounds, sugars, polyphenol contents, and antioxidant activity data of control (Cb) and started (Sb) non-alcoholic einkorn-based fermented beverages.

PCA (Figure 3) was performed using data related to organic acids, volatile compounds, sugars, TPC, and antioxidant activity to evaluate differences and/or similarities between control and started prototypes. The sum of the first two principal components explained 74.8% of the total variance. PC1 and PC2 represented 48.3% and 26.5% of the total variance, respectively. The fermented non-alcoholic einkorn-based beverages were classified into three clusters (Figure 3a). According to PC1, Sb_t0_, Cb_t0_, Cb_t1_, and Cb_t29_ were positioned on the negative axis, while Sb_t1_ and Sb_t29_ were positioned on the positive axis. PC2 distributed Cb_t1_ and Cb_t29_ on the positive axis, while the Cb_t0_ and Sbt_0_ were positioned on the negative axis.

**Figure 4:**
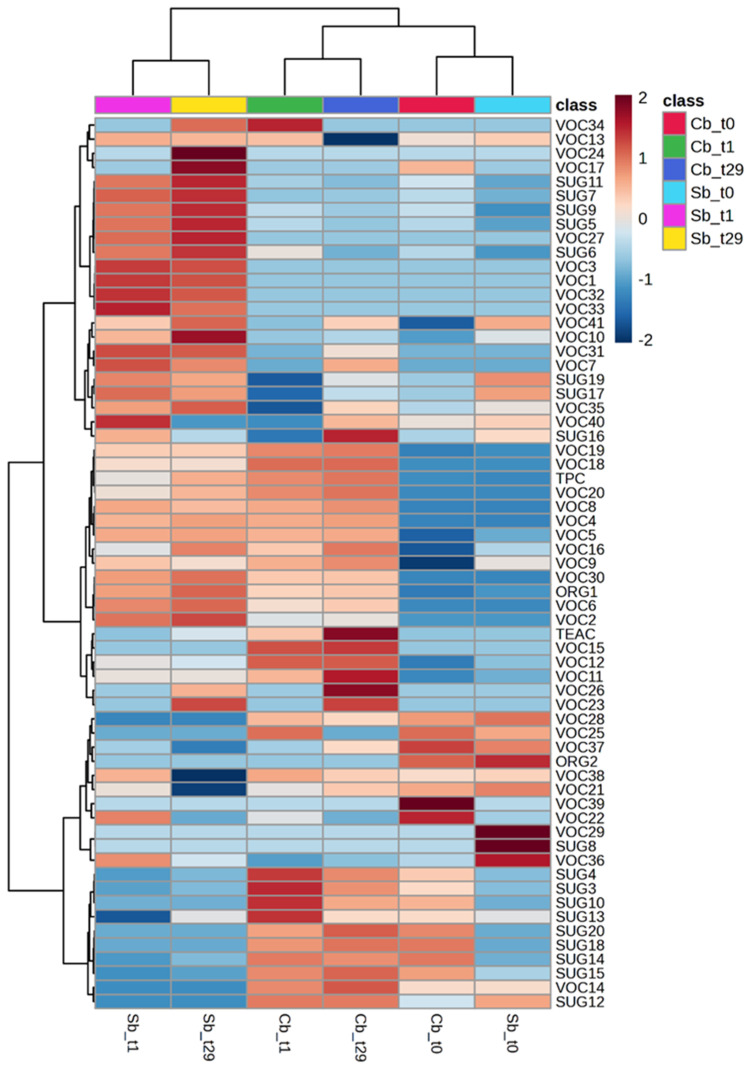
Heatmap analysis was carried out using all concentrations of variables such as organic acids, volatile compounds, sugars, polyphenol contents, and antioxidant activity evaluated in non-started controls (including Cbt_0_, Cbt_1_, and Cbt_29_) and started prototypes (including Sbt_0_, Sbt_1_, and Sbt_29_) of non-alcoholic einkorn-based fermented beverages at different sampling times.

The loading plot, depicted in Figure 3b, displays the relative relevance of variables. A heatmap (Figure 4) was also generated to represent all variables, providing further support for the findings from the PCA analysis.

The higher content in certain sugars, such as pyranose (SUG3), furanose (SUG4 and SUG10), mannitol (SUG12), and disaccharides (SUG15, SUG18, SUG20), resulted as the variables differentiating the cluster consisting of Cb_t1_ and Cb_t29_ from the other two groups. Additionally, heptanol (VOC15) was characteristic of this cluster (Figure 3b and Figure 4). β-Fructopyranose (SUG7), β-glucose (SUG11), α-fructofuranose (SUG5), α-glucose (SUG9), and β-fructofuranose (SUG6) were the variables that determined the clusterization of Sb_t1_ and Sb_t29_. Furthermore, the VOCs isobutenylcarbinol (VOC10) and 2-nonanone (VOC31) were characteristic of this cluster, while 2-heptenal (VOC24) was only characteristic of Sb_t29_.

The clustering of Sb_t0_ and Cb_t0_ was attributed to the lower levels of certain volatile compounds in the stored samples, specifically isovaleric acid (VOC5) and octanol (VOC19). Other identified VOCs, such as 1-nonanol (VOC20), butyric acid (VOC4), and ethanol (VOC8), were absent in both Sb_t0_ and Cb_t0_. Moreover, all samples exhibited an increase in lactic acid (ORG1) and TPC after storage.

### 3.8. Proximate Composition and Energy Content of Control and Started Prototypes

The results of the proximate composition analysis of the fermented einkorn-based beverages after fermentation are reported in Table 7. Overall, no statistically significant differences were observed between started and control samples. The main component was water (~91 g 100 g^−1^), followed by carbohydrates (~7.58 g 100 g^−1^), proteins (~0.62 g 100 g^−1^), dietary fiber (~0.38 g 100 g^−1^), ash (~0.11 g 100 g^−1^), and lipid (~0.03 g 100 g^−1^). Analogously, the energy content was not statistically significantly different between the prototypes, resulting in 33.59 ± 0.48 Kcal 100 g^−1^ in started beverages and 34.01 ± 3.62 Kcal 100 g^−1^ in control beverages.

The results herein collected are in agreement with the data reported by Cardinali et al. [15] on red rice-, barley-, and buckwheat-based beverages prepared using a very similar technological process. Although cereal- and pseudocereal-based beverages are generally considered as good sources of fibers and proteins [32], the steps involved in the manufacturing process (i.e., filtration and dilution) might be responsible for the low concentration of fibers and protein.

### 3.9. Sensory Attributes of Started Prototypes

**Figure 5:**
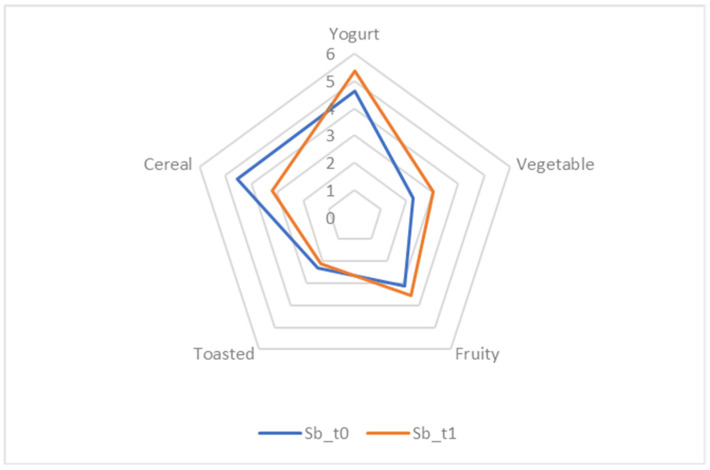
A spider plot reporting the data of the sensory analysis performed on the started prototypes of the non-alcoholic einkorn-based fermented beverage before (Sbt_0_) and after (Sbt_1_) the fermentation.

The results of the sensory evaluation of the olfactory attributes of the started prototypes at t_0_ and t_1_ are depicted in Figure 5. No statistically significant differences were observed between the score values obtained for the beverage before and after fermentation. Additionally, the overall acceptability was also similar for both samples, with scores of 5.85 ± 1.55 (Sb_t0_) and 4.77 ± 2.46 (Sb_t1_). After fermentation, the started prototype was characterized by a higher perception of yogurt and fruity attributes compared to the beverage before fermentation. Moreover, the score for “cereal” odor was more pronounced at t_0_ than after fermentation.

The yogurt and fruity notes perceived by the sensory analysis are characteristic of fermented products. These attributes can be associated with volatile compounds mainly derived from microbial activity. Specifically, acetoin, 2-heptanone, 2,3-butanediol, butyric acid, and caproic acid may confer buttery, cheesy, creamy, acidic, and waxy notes, while 2-nonanone is mostly related to fruity perceptions. Acetoin and 2-heptanone were previously detected by Coda et al. [25] in vegetable yogurt-like beverages made with rice, soy, barley, emmer, and oat flour. Thus, the fermentation of einkorn wheat could potentially enhance the plant-based beverage aroma.

## 4. Conclusions

The screening of twenty lactic acid bacteria isolates, in monoculture and in mini-batch fermentations in an ad hoc-prepared einkorn-based substrate, allowed for the selection of the strains with the most suitable technological properties for the fermentation of a novel cereal-based non-alcoholic beverage. The formulated multiple-strain starter (BZ33, BZ34, BZ47), indeed, determined a fast acidification after 24 h fermentation and remained viable during 29 days of refrigerated storage, thereby ensuring the safety and stability of the beverage. Furthermore, the fermentative metabolism of lactic acid bacteria yielded volatile compounds typical of fermented products, contributing yogurt and fruity notes to the beverage’s sensory profile. Total polyphenol content increased during fermentation and remained stable during storage, whereas antioxidant activity did not show significant differences over time. However, to obtain a more precise evaluation of antioxidant activity, the use of DPPH and ABTS tests is advisable. These findings suggest the possibility of producing a non-alcoholic einkorn-based fermented beverage that is safe and stable over time, with pleasant sensory properties. A further characterization analysis might be performed in the future to comprehensively evaluate the nutritional value of this novel non-alcoholic cereal-based fermented beverage.

## Figures and Tables

**Table 1:** Results of pH measurements after 8 h and 24 h mini-batch fermentations of the einkorn-based substrate.

Strain	Species	pH (8 h)	pH (24 h)
BZ2	*Limosilactobacillus fermentum* (basonym *Lactobacillus fermentum*)	5.93 ± 0.04 ^efgh^	4.27 ± 0.03 ^efg^
BZ5	*Limosilactobacillus fermentum* (basonym *Lactobacillus fermentum*)	6.09 ± 0.04 ^def^	4.26 ± 0.05 ^fg^
BZ10	*Lacticaseibacillus paracasei* (basonym *Lactobacillus paracasei*)	7.04 ± 0.08 ^a^	4.02 ± 0.04 ^gh^
BZ21	*Lacticaseibacillus casei* (basonym *Lactobacillus casei*)	5.87 ± 0.03 ^efgh^	4.05 ± 0.04 ^gh^
BZ22	*Lacticaseibacillus paracasei* (basonym *Lactobacillus paracasei*)	6.68 ± 0.11 ^b^	5.05 ± 0.12 ^bc^
BZ26	*Lentilactobacillus parabuchneri* (basonym *Lactobacillus parabuchneri*)	5.98 ± 0.04 ^defgh^	4.74 ± 0.06 ^cd^
BZ28	*Lentilactobacillus parabuchneri* (basonym *Lactobacillus parabuchneri*)	6.15 ± 0.19 ^cde^	4.79 ± 0.04 ^cd^
BZ30	*Lentilactobacillus parabuchneri* (basonym *Lactobacillus parabuchneri*)	6.25 ± 0.01 ^cd^	4.73 ± 0.06 ^cd^
BZ31	*Lentilactobacillus parabuchneri* (basonym *Lactobacillus parabuchneri*)	6.45 ± 0.05 ^bc^	4.67 ± 0.01 ^cde^
BZ32	*Lentilactobacillus buchneri* (basonym *Lactobacillus buchneri*)	6.42 ± 0.07 ^bc^	4.73 ± 0.07 ^cd^
BZ33	*Lacticaseibacillus casei* (basonym *Lactobacillus casei*)	5.68 ± 0.03 ^hi^	3.71 ± 0.01 ^h^
BZ34	*Lacticaseibacillus casei/paracasei* (basonym *Lactobacillus casei/paracasei*)	5.73 ± 0.04 ^ghi^	3.85 ± 0.03 ^h^
BZ35	*Lacticaseibacillus casei (basonym Lactobacillus casei)*	7.27 ± 0.14 ^a^	5.26 ± 0.04 ^b^
BZ36	*Lentilactobacillus parabuchneri* (basonym *Lactobacillus parabuchneri*)	6.04 ± 0.03 ^defg^	4.54 ± 0.23 ^def^
BZ37	*Lentilactobacillus parabuchneri* (basonym *Lactobacillus parabuchneri*)	6.05 ± 0.01 ^def^	5.22 ± 0.11 ^b^
BZ38	*Lentilactobacillus parabuchneri* (basonym *Lactobacillus parabuchneri*)	5.91 ± 0.04 ^efgh^	4.58 ± 0.14 ^def^
BZ39	*Pediococcus parvulus*	7.30 ± 0.14 ^a^	5.91 ± 0.27 ^a^
BZ43	*Loigolactobacillus coryniformis* (basonym *Lactobacillus coryniformis*)	5.67 ± 0.08 ^hi^	4.03 ± 0.04 ^gh^
BZ44	*Loigolactobacillus coryniformis* (basonym *Lactobacillus coryniformis*)	5.82 ± 0.06 ^fghi^	4.04 ± 0.04 ^gh^
BZ47	*Lacticaseibacillus casei/paracasei* (basonym *Lactobacillus casei/paracasei*)	5.55 ± 0.02 ^i^	3.78 ± 0.03 ^h^

The results are expressed as mean values of three replicates ± standard deviations. For each sampling time, values labeled with different letters in the same column are significantly different (*p* ≤ 0.05).

**Table 2:** Results of microbial viable counts of presumptive mesophilic lactobacilli, mesophilic aerobic bacteria, spore-forming bacteria, yeasts, and Enterobacteriaceae performed on control (Cb) and started (Sb) prototypes of non-alcoholic einkorn-based fermented beverages.

Microbial Group	Sampling Time	Prototypes	
Presumptive mesophilic lactobacilli		Cb	Sb
	t_0_	<1.0 ^b,B^	6.3 ± 0.0 ^c,A^
	t_1_	5.1 ± 0.4 ^a,B^	9.0 ± 0.0 ^b,A^
	t_8_	4.0 ± 0.8 ^a,B^	9.0 ± 0.1 ^ab,A^
	t_15_	4.2 ± 0.8 ^a,B^	9.1 ± 0.0 ^a,A^
	t_22_	4.5 ± 0.7 ^a,B^	9.2 ± 0.1 ^a,A^
	t_29_	4.5 ± 0.8 ^a,B^	9.1 ± 0.0 ^ab,A^
Mesophilic aerobic bacteria		Cb	Sb
	t_0_	3.1 ± 0.4 ^b,B^	6.3 ± 0.0 ^b,A^
	t_1_	5.6 ± 0.3 ^a,B^	9.0 ± 0.0 ^a,A^
	t_8_	4.6 ± 0.7 ^ab,B^	9.0 ± 0.1 ^a,A^
	t_15_	5.1 ± 0.7 ^a,B^	9.1 ± 0.1 ^a,A^
	t_22_	4.7 ± 1.0 ^ab,B^	9.1 ± 0.1 ^a,A^
	t_29_	4.7 ± 0.7 ^ab,B^	9.1 ± 0.0 ^a,A^
Spore-forming bacteria		Cb	Sb
	t_0_	2.0 ± 0.6 ^b,A^	2.0 ± 0.5 ^a,A^
	t_1_	4.5 ± 0.7 ^a,A^	3.6 ± 0.7 ^a,A^
	t_8_	4.0 ± 0.6 ^ab,A^	3.7 ± 0.7 ^a,A^
	t_15_	3.8 ± 1.0 ^ab,A^	3.6 ± 0.7 ^a,A^
	t_22_	3.5 ± 0.8 ^ab,A^	3.6 ± 0.7 ^a,A^
	t_29_	3.2 ± 0.8 ^ab,A^	3.6 ± 0.7 ^a,A^
Yeasts		Cb	Sb
	t_0_	<1.0	<1.0
	t_1_	<1.0	<1.0
	t_8_	<1.0	<1.0
	t_15_	<1.0	<1.0
	t_22_	<1.0	<1.0
	t_29_	<1.0	<1.0
Enterobacteriaceae		Cb	Sb
	t_0_	<1.0	<1.0
	t_1_	<1.0	<1.0
	t_8_	<1.0	<1.0
	t_15_	<1.0	<1.0
	t_22_	<1.0	<1.0
	t_29_	<1.0	<1.0

The results are expressed as mean Log CFU g^−1^ of three replicates ± standard deviations. Values labeled with different lowercase letters in the same column are significantly different (*p* ≤ 0.05), whereas values labeled with different capital letters in the same row are significantly different (*p* ≤ 0.05).

**Table 3:** Total phenolic content and antioxidant capacity of non-started control (Cb) and started prototype (Sb) of non-alcoholic einkorn-based fermented beverages at different sampling times.

Sampling Time	Prototype	TPC (mg GAE kg ^−1^)	FRAP (μM TEAC)
t_0_	Cb	13.33 ± 0.77 ^c^	84.56 ± 3.89 ^b^
	Sb	13.00 ± 1.41 ^c^	82. 58 ± 7.35 ^b^
t_1_	Cb	36.26 ± 4.26 ^a^	100.31 ± 18.02 ^b^
	Sb	24.29 ± 5.69 ^b^	83.58 ± 14.82 ^b^
t_29_	Cb	38.47 ± 3.67 ^a^	166.83 ± 22.63 ^a^
	Sb	32.37 ± 6.71 ^a^	88.57 ± 6.32 ^b^

Results are expressed as mg GAE kg^−1^ and μM TEAC on fresh weight. Different lowercase letters in the same column indicate significant differences (*p* ≤ 0.05).

**Table 6:** Concentration of organic acids, sugars (mono- and disaccharides), and polyalcohols of the control (Cb) and started (Sb) non-alcoholic einkorn-based fermented beverages at different sampling times. Results are reported as mg kg^−1^.

Nº	Rt	Compound	Cb_t0_	Cb_t1_	Cb_t29_	Sb_t0_	Sb_t1_	Sb_t29_
Organic acids								
1	8.23	Lactic acid	33.2 ± 14.2 ^d^	955.2 ± 486.4 ^c^	1098.5 ± 111.7 ^c^	46.2 ± 8.9 ^d^	2168.9 ± 213.4 ^b^	5025.1 ± 438.8 ^a^
2	14.54	Malic acid	9.5 ± 8.9 ^a^	n.d.	n.d.	11.6 ± 1.4 ^a^	n.d.	n.d.
		Total	44.1 ± 6.4 ^d^	955.2 ± 486.4 ^c^	1098.5 ± 111.7 ^c^	57.8 ± 8.8 ^d^	2168.9 ± 213.4 ^b^	5025.1 ± 438.8 ^a^
Monosaccharides								
3	17.09	(pyranose)	177.7 ± 117.5 ^b^	1122.4 ± 199.7 ^a^	387.8 ± 140.0 ^b^	28.4 ± 8.5 ^b^	18.9 ± 8.3 ^b^	26.1 ± 4.1 ^b^
4	17.69	(furanose)	260.3 ± 162.2 ^bc^	855.4 ± 57.1 ^a^	449.4 ± 182.3 ^b^	47.4 ± 14.9 ^c^	31.6 ± 16.4 ^c^	43.5 ± 13.5 ^c^
5	18.16	α-Fructofuranose	73.7 ± 35.3 ^c^	83.2 ± 55.1 ^c^	44.4 ± 16.4 ^c^	31.3 ± 22.5 ^c^	750.9 ± 171.2 ^b^	1693.4 ± 249.5 ^a^
6	18.25	β-Fructofuranose	99.9 ± 29.7 ^c^	199.8 ± 27.5 ^c^	49.6 ± 1.8 ^c^	36.9 ± 21.2 ^c^	941.3 ± 226.3 ^b^	1899.1 ± 181.1 ^a^
7	18.31	β-Fructopyranose	118.7 ± 64.8 ^c^	69.4 ± 57.4 ^c^	73.7 ± 2.8 ^c^	53.1 ± 29.0 ^c^	1589.4 ± 34.7 ^b^	2914.5 ± 295.5 ^a^
8	18.73	α-Galactose	n.d.	n.d.	n.d.	5.6 ± 1.2	n.d.	n.d.
9	19.10	α-Glucose	496.3 ± 189.8 ^c^	563.5 ± 238.2 ^c^	344.5 ± 7.7 ^c^	146.1 ± 35.1 ^c^	2414.0 ± 257.7 ^b^	4466.9 ± 234.1 ^a^
10	19.77	(furanose)	57.3 ± 43.8 ^ab^	243.0 ± 102.8 ^a^	56.3 ± 32.9 ^b^	n.d.	n.d.	n.d.
11	19.92	β-Glucose	626.8 ± 218.7 ^c^	565.4 ± 298.9 ^c^	294.3 ± 65.3 ^c^	259.1 ± 135.5 ^c^	2820.0 ± 268.3 ^b^	5353.7 ± 618.0 ^a^
		Total	1883.5 ± 207.1 ^cd^	3558.1 ± 610.0 ^c^	1480.8 ± 368.9 ^d^	609.6 ± 216.0 ^d^	8566.1 ± 972.3 ^b^	16,397.2 ± 1060.3 ^a^
Polyalcohols								
12	19.46	Mannitol	3.6 ± 2.8 ^c^	60.8 ± 3.1 ^a^	61.5 ± 18.0 ^a^	26.8 ± 8.5 ^b^	n.d.	n.d.
13	21.04	Myo-inositol	50.6 ± 26.6 ^a^	44.1 ± 12.0 ^a^	36.0 ± 6.8 ^a^	24.7 ± 8.8 ^a^	26.6 ± 5.4 ^a^	36.5 ± 17.0 ^a^
		Total	53.3 ± 25.3 ^bc^	104.9 ± 8.9 ^a^	97.5 ± 24.7 ^abc^	53.9 ± 19.2 ^bc^	26.6 ± 5.4 ^c^	36.5 ± 17.0 ^c^
Disaccharides								
4	24.68	Sucrose	1058.8 ± 434.3 ^a^	997.2 ± 387.6 ^a^	776.2 ± 199.1 ^a^	48.2 ± 25.0 ^b^	29.2 ± 5.1 ^b^	49.8 ± 21.3 ^b^
15	25.49	(disaccharide)	339.7 ± 122.0 ^ab^	383.6 ± 174.9 ^ab^	457.4 ± 187.0 ^a^	161.5 ± 147.1 ^ab^	74.3 ± 11.5 ^b^	90.5 ± 35.4 ^b^
16	25.64	Saccharose	15,986.2 ± 1709.4 ^bc^	14,590.8 ± 1170.5 ^b^	19,378.6 ± 892.2 ^a^	17,061.8 ± 826.0 ^ab^	17,693.6 ± 313.1 ^acd^	16033.1 ± 947.7 ^bd^
17	25.94	Maltose	438.7 ± 56.4 ^b^	249.2 ± 75.3 ^b^	533.0 ± 238.6 ^b^	960.5 ± 214.2 ^a^	1155.6 ± 119.0 ^a^	955.6 ± 121.5 ^a^
18	26.17	(disaccharide)	594.1 ± 149.5 ^a^	476.5 ± 139.0 ^a^	597.3 ± 73.0 ^a^	n.d.	n.d.	n.d.
19	26.34	Maltose	531.3 ± 62.1 ^cd^	284.9 ± 124.5 ^d^	781.9 ± 335.7 ^cb^	1364.6 ± 335.1 ^a^	1391.5 ± 124.8 ^a^	1190.9 ± 111.3 ^ab^
20	26.76	(disaccharide)	725.0 ± 61.0 ^a^	616.2 ± 196.3 ^a^	1005.2 ± 214.1 ^a^	n.d.	n.d.	n.d.
		Total	19,673.7 ± 2166.9 ^ab^	17,598.3 ± 1725.0 ^b^	23,529.7 ± 1911.4 ^a^	19,592.5 ± 1181.7 ^ab^	20,344.2 ± 551.8 ^ab^	18,976.6 ± 368.2 ^b^

Different letters in the same row indicate statistically significant differences among samples (*p* ≤ 0.05). n.d.: not detected.

**Table 7:** Proximate composition of control (Cb) and started (Sb) laboratory-scale prototypes of non-alcoholic einkorn-based fermented beverages.

Sample	Moisture	Dry Matter	Carbohydrate	Protein	Lipid	Dietary Fiber	Ash
Cb	90.77 ± 1.04 ^a^	9.23 ± 1.04 ^a^	7.62 ± 0.64 ^a^	0.64 ± 0.19 ^a^	0.02 ± 0.01 ^a^	0.40 ± 0.17 ^a^	0.11 ± 0.02 ^a^
Sb	91.17 ± 0.36 ^a^	8.83 ± 0.36 ^a^	7.55 ± 0.15 ^a^	0.60 ± 0.04 ^a^	0.03 ± 0.01 ^a^	0.36 ± 0.01 ^a^	0.10 ± 0.00 ^a^

Results are expressed as g 100 g^−1^. Values labeled with different lowercase letters in the same column are significantly different (*p* ≤ 0.05).

## Data Availability

Original contributions presented in the study are included in the article; further inquiries can be directed to the corresponding author.
